# The Microbiome and Radiation Induced-Bowel Injury: Evidence for Potential Mechanistic Role in Disease Pathogenesis

**DOI:** 10.3390/nu10101405

**Published:** 2018-10-02

**Authors:** Tomoko Kumagai, Farooq Rahman, Andrew M. Smith

**Affiliations:** 1 UCL Eastman Dental Institute, University College London (UCL), Rayne Institute, 5 University Street, London WC1E 6JF, UK; andrew.m.smith@ucl.ac.uk; 2 Department of Gastroenterology, University College London Hospitals NHS Foundation Trust, 250 Euston Road, London NW1 2PG, UK; farooq.rahman@nhs.net

**Keywords:** radiation-induced bowel injury, radiation enteritis, radiation enteropathy, pelvic radiation disease, radiotherapy, cancer management complications, microbiota, probiotics

## Abstract

Radiotherapy has played a major role in both the curative and palliative treatment of cancer patients for decades. However, its toxic effect to the surrounding normal healthy tissue remains a major drawback. In cases of intra-abdominal and/or pelvic malignancy, healthy bowel is inevitably included in the radiation field, causing undesirable consequences that subsequently manifest as radiation-induced bowel injury, which is associated with significant morbidity and mortality. The pathophysiology of radiation-induced bowel injury is poorly understood, although we now know that it derives from a complex interplay of epithelial injury and alterations in the enteric immune, nervous, and vascular systems in genetically predisposed individuals. Furthermore, evidence supporting a pivotal role for the gut microbiota in the development of radiation-induced bowel injury has been growing. In this review, we aim to appraise our current understanding of radiation-induced bowel injury and the role of the microbiome in its pathogenesis as well as prevention and treatment. Greater understanding of the relationship between the disease mechanism of radiation-induced bowel injury and gut microbiome might shed light on potential future prevention and treatment strategies through the modification of a patient’s gut microbiome.

## 1. Introduction

Cancer has been the disease of this century, with significant increases in both incidence and prevalence. The current worldwide estimate of new cases of cancer is 14 million annually, which is expected to rise above 23 million by 2030 [1]. Radiotherapy has been a vital part of the therapeutic armamentarium for both the curative and palliative management of cancer patients over the last few decades. Despite advances in radiation physics and technology, collateral damage to the surrounding healthy tissue remains a major complication of radiation therapy.

In the United Kingdom (U.K.), there were almost 350,000 new cases of cancer in 2015. More than 36% originated from organs within the abdomen, retroperitoneum, or pelvis, and it is estimated that approximately 35,000 patients underwent radiotherapy directed to their abdominopelvic region [2]. The radiation field for abdominopelvic cancers inevitably includes relatively significant parts of healthy intestine, which is an organ that is particularly susceptible to radiation toxicity, causing both acute and chronic injury to the intestine manifesting as radiation-induced bowel injury.

Despite increased awareness of the significant morbidity and mortality associated with radiation-induced bowel injury, our understanding of its pathophysiology and consequently the treatment options remain suboptimal [3]. It is becoming increasingly clear that radiation-induced bowel injury derives from a complex interplay of epithelial injury, and alterations in the enteric immune, nervous, and vascular systems. A genetic predisposition of individuals to radiosensitivity has also been implicated in the disease process [4]. Furthermore, supported by a recent advance in technology such as next generation sequencing with 16S rRNA gene amplicon analysis, there is emerging evidence implicating the microbiome in the pathogenesis of radiation-induced bowel injury. The latest evidence also suggests that the influence of environmental factors on the microbiome is much greater than an individual’s genetic background [5,6]. From this key evidence, it can be proposed that manipulating the microbiome by changing environmental factors may offer potential therapeutic benefits.

As cancer survival rates improve, a trend that is expected to continue, more people than ever are living longer having received cancer therapies. As approximately 50% of current cancer survivors received radiotherapy for cancers arising within the abdominopelvic cavity, there will be a significant increase in the global burden of radiation-induced bowel injury in the future [7]. Therefore, a better understanding of the disease mechanism underlying this debilitating yet relatively discounted disorder is urgently needed in order to improve patient care as well as develop preventative treatment. In this review, we will provide an overview of our current understanding of radiation-induced bowel injury and how the gut microbiome may play a pivotal role in the development, prevention, and treatment of this disease.

## 2. Clinical Significance of Radiation-Induced Bowel Injury

**Figure 1:**
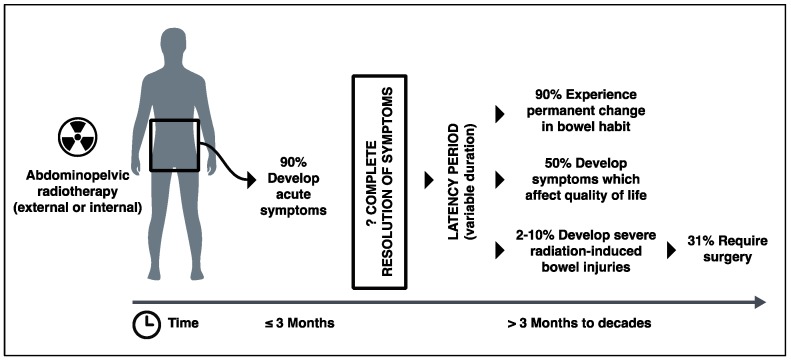
Clinical course of patients undergoing radiotherapy to abdominopelvic region and consequential development of radiation-induced bowel injury over time.

Due to the lack of consensus about diagnostic criteria and an under-reporting of symptoms by patients, the true incidence of radiation-induced bowel injury is unknown. However, it is said that up to 90% of patients experience gastrointestinal symptoms in the first few weeks after receiving radiation to their abdominopelvic region [3,7,8]. There is also evidence to suggest that radiooncological inpatients have high incidence of *Clostridium difficile* infection with high mortality [9]. Although there are discrepancies between the figures in the literature, up to 90% of patients go on to experience some form of permanent change in their bowel habit, and at least 50% of patients report that their chronic gastrointestinal symptoms significantly affect their quality of life [3,10,11,12] (Figure 1). Yet, how many long-term gastrointestinal symptoms are truly due to progressive damage to the bowel caused by radiation is difficult to determine. Many studies do not exclude conditions such as infection, small bowel bacterial overgrowth, and bile acid malabsorption (which is common after abdominopelvic radiation exposure), nor take account of confounding factors such as medications and co-morbidities that could potentially cause patents’ gastrointestinal symptoms. Nevertheless, a number of studies suggest that 3–10% of patients who undergo abdominopelvic radiotherapy develop a severe form of radiation-induced bowel injury with evidence of dysmotility, strictures, fibrosis, and fistulae [3,13,14,15]. One-third of these patients with severe radiation-induced bowel injury will require surgery that is associated with a direct mortality rate of 10–22% [14,16,17] (Figure 1).

Typical symptoms of radiation-induced bowel injury include diarrhea, abdominal pain, bloating, urgency, flatulence, fecal incontinence, bleeding per rectum, and weight loss. “Acute” symptoms occur within three months of the commencement of radiotherapy and usually resolve within three months. However, some authors argue otherwise, and exactly how many of this heterogeneous cohort of patients, with various cancer types, radiotherapy regimes, and symptoms become symptom-free and how many continue to have symptoms beyond three months is unclear [18]. Symptoms that start more than three months after radiotherapy are regarded as “chronic” in clinical settings, and although they typically manifest between six months to three years after initial radiotherapy, a latency period up to 20 years is not unusual. Manifestations of chronic radiation-induced bowel injury include strictures and bowel obstruction, perforation, fistulae, and abscess formation and malabsorption.

It has been reported that the degree of tissue damage correlates with the intensity of the radiation dose, dosing schedule, mode of radiation delivery and the volume of bowel contained in the radiation field [19]. Other risk factors include the concurrent use of chemotherapy, pre-existing inflammatory bowel disease, and other co-morbidities (such as vascular disease and HIV), as well as predisposing genetic factors [19,20].

## 3. Pathophysiology of Radiation-Induced Bowel Injury

### 3.1. Cellular Effect of Radiation

Ionising radiation carries enough energy to release electrons from atoms or molecules that are then capable of cell damage and death by several mechanisms. Detailed review articles of the effect of radiation on the biology of cells have been written by Riley [21] and Azzam [22].

Briefly, the energy carried by radiation emissions can physically disrupt DNA structures, leading to single and double-strand breaks, and sugar or base damage [23]. Direct DNA damage caused by ionising radiation occurs in clusters within the DNA structure (which is more resistant to a cell’s own DNA repair mechanism) [24].

Secondly, ionizing radiation causes the radiolysis of water (70% of a cell’s makeup) and stimulates nitrogen oxide synthetase to produce reactive oxygen species (ROS) and reactive nitrogen species (NOS), respectively. Radiation also causes electron leakage from mitochondria, the power house of the cell producing ATP by aerobic respiration, which leads to the production of a powerful ROS, superoxide [25]. ROS and NOS are normally produced by cells and have important biological functions such as ROS production in defense against microbes. Overall, the toxic effects of these molecules include DNA/RNA damage as well as amino acid oxidation and lipid peroxidation, resulting in nucleic acid damage, mutation, and protein and lipid disruption within the cell [26,27].

The above events result in the disruption of the cell homeostasis, the activation of pro-inflammatory signaling pathways, notably via NF-κB, cell cycle arrest and cell death by apoptosis (by activation of p53), or necrosis.

### 3.2. Radiation-Induced Acute Bowel Injury

Generally speaking, radiosensitive cells are undifferentiated, rapidly dividing, well-nourished, and have high metabolic activity [28,29]. In both small and large bowel, undifferentiated intestinal stem cells are located within the crypts of the mucosal surface. Stem cells migrate upwards to the tips of villi, and upon doing so differentiate into dedicated epithelial cells, such as enterocytes, goblet cells, and enteroendocrine cells, before undergoing apoptosis and being shed. During this maturation process, particularly at the transitional amplifying stage, cells proliferate rapidly dividing every 12–18 h, providing a pool of progenitors, before fully committing to the final epithelial cell lineage [30]. Epithelial cells in the small intestinal and colonic villi are reported to have turnover rates of 1.5 days and 4.2 days, respectively, making them the most rapidly proliferating fixed tissue cells in the entire body [31]. The high proliferative rate of intestinal epithelial cells, particularly intestinal stem cells, makes the bowel one of the most susceptible organs to radiation injury.

**Figure 2:**
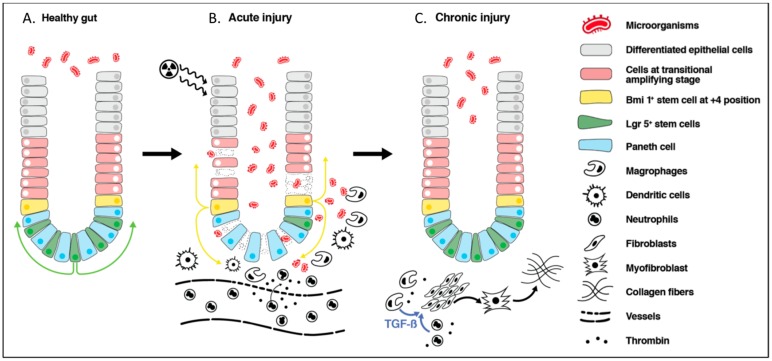
Schematic representation of radiation-induced intestinal injury. (**A**): In healthy gut, crypts are sterile with intact mucosa. Lgr5^+^ stem cells proliferate and cells migrate upwards to provide differentiated epithelial cells of the villi. Bmi1^+^ stem cells located at the +4 position remain quiescent. (**B**): In the acute phase of radiation-induced injury, there is loss of epithelial barrier integrity, leading to an influx of antigenic material, including microorganisms into the lamina propria. This induces inflammation orchestrated by macrophages, dendritic cells, and recruited neutrophils from the circulation. Further inflammation is caused by endothelial cell damage and the release of thrombin. Mitotically active Lgr5^+^ cells undergo apoptosis where as Bmi1^+^ cells at the +4 position are resistant to radiation insult and acquire an Lgr5^+^ phenotype to act as a stem cell reservoir. (**C**): Despite epithelial barrier restoration, during the chronic phase of injury, there is an increase in the TGF-β that is secreted by macrophages and neutrophils, which promotes the differentiation of the fibroblast into myofibroblast, leading to collagen deposition and fibrogenesis.

Although stem cells and the epithelial cells of the villi are the most radiosensitive, the intestine as an organ is comprised of many different cell types, including enteric immune, vascular, and nervous systems, which are all affected by irradiation to various degrees. The overall acute consequence of radiation to the bowel is reduced tight junction integrity, the death of crypt epithelial cells, as well as those higher up the villi, depending on the intensity of the radiation [32,33,34]. These effects can lead to the development of inflammation and breakdown in the mucosal barrier, enabling an influx of luminal contents, particularly microorganisms, into the lamina propria, triggering further inflammation and the recruitment of immune cells [35,36] (Figure 2). In addition, endothelial cell damage resulting from radiotherapy has been shown to contribute to mucosal inflammation [37]. Disruption in vascular architecture leads to microhemorrhage and cascades of prothrombotic and pro-inflammatory events, causing further inflammation as well as vascular occlusion and reduced blood supply to the site of mucosal injury [38]. An acute reversible alteration in small intestine motility has also been shown in animal studies [39,40].

These acute effects of radiation occur immediately after the radiotherapy and become clinically evident within days.

### 3.3. Epithelial Cell Recovery

The majority of acute symptoms subside within a few weeks of completion of radiotherapy as the stem cells regenerate to reform the protective epithelial barrier. In fact, the entire crypt–villus structure can be repaired in six to eight days, even if only a single stem cell survived radiotherapy [41]. Current evidence suggests that stem cells in the intestinal crypts are composed of cells of different activity and possibly functional states [42,43]. Lgr5^+^ stem cells are mitotically active cells that divide constantly and migrate up the villi, maturing into epithelial cells. These cells are highly sensitive to radiation, which induces apoptosis [43,44]. Bmi1^+^ stem cells located at the +4 position of the crypt, on the other hand, are quiescent and more radioresistant [43,45]. They are thought to act as a stem cell reservoir within the intestine in the event of tissue damage resulting from infection, inflammation, or radiation injury [41,46] (Figure 2). Furthermore, the evidence suggests that there is an increased recruitment of mesenchymal stem cells to the intestinal epithelium following radiation injury [47]. This seems to promote and accelerate the structural integrity of the intestinal epithelium [48]. If the entire stem cell population is lost within crypts over a larger surface area, then this leads to a prolonged repair process and potentially results in intestinal failure. As radiotherapy usually involves multiple sessions of radiation application, the recovery process is halted each time, with cumulative toxic effects prolonging the inflammatory state and recovery duration.

### 3.4. Radiation-Induced Chronic Bowel Injury

An early histological examination of resected bowel specimens with chronic radiation-induced lesions identified fibrosis, obliterating vasculitis, atrophy, or the degeneration of muscles fibers as well as morphological changes in fibroblasts, endothelial, and epithelial cells as the characteristic features of this pathology [49]. Although the restoration of the epithelial layer is thought to occur within a few weeks of the completion of radiotherapy along with the cessation of symptoms, the subclinical repair process of the intestine seems to last much longer with irreversible consequences in some patients. An increase in the expression of TGF-β1 which is a potent profibrogenic cytokine, is seen in epithelial cells, lamina propria, submucosa, subserosa, and smooth muscle cells soon after radiation. Although expression returns to baseline in epithelial cells, it remains elevated in fibroblasts in lamina propria, endothelial cells, and smooth muscle at week 26 post-irradiation [50]. The marked upregulation of collagen and both matrix metalloproteases (MMP) (fibrolytic) and the tissue inhibitor of metalloproteinase (TIMP) (fibrogenic) were also found in human bowel resection specimens with chronic radiation-induced injury [51]. The increase in MMP was proportional to the extent of inflammatory cell infiltration, demonstrating a dynamic tissue repair process that continues over a long period of time. An increase in the macrophage and neutrophil populations in the lamina propria is thought to participate in the fibrosis of the intestine. The secretion of cytokines such as TGF-β1 from macrophages and neutrophils drives fibroblast–myofibroblast and the deposition of the extracellular matrix and fibrogenesis [52]. Thrombin, which is activated by vascular damage, plays a central role in clotting formation, and also stimulates various cells, including leukocytes, fibroblasts, endothelial cells, and smooth muscle cells, contributing to fibrogenesis and inflammation [38] (Figure 2). Furthermore, a number of animal studies have reported the altered secretion of neurotransmitters within the enteric nervous system and reduced motility [53,54]. One of the few studies with human tissue samples showed a reduction in the degree of the vasoactive intestinal peptide (VIP) and Substance P (SP) intestinal innervation in the specimens taken a few weeks to months following abdominopelvic radiotherapy [55].

Overall, the chronic phase of enteropathy is characterized by chronic inflammation, fibrosis, progressive occluding vasculitis, ischemia, and dysmotility. The relationship between acute and delayed injury as well as the reasons behind the prolonged latency period of delayed injury in some patients are still unclear.

## 4. Microbiome and Radiation-Induced Bowel Injury

### 4.1. Importance of Microbiome in Healthy Gut

Gut microbiota are a community of microorganisms that populate the luminal space and mucosal surface of our gastrointestinal tract. The gut microbiome refers to the entire genetic material harbored by gut microbiota. However, the two terms are often used interchangeably. Each human carries up to 10^14^ microbes, collectively weighing approximately 2 kg, or more than the weight of the brain. The composition of microbiota is unique to an individual, but it is not fixed and can alter in response to a number of factors such as changes in the environment, medication, and health. The vast majority of the organisms that make up gut microbiota are bacteria; however, viruses, fungi, archaea, and protists are also present. Colonization of the human gastrointestinal tract with microorganisms starts immediately after birth, and is heavily influenced by the mode of delivery [56]. By the age of three, the profile of microbiota of an individual becomes established and stays fairly stable throughout the remainder of life [57,58]. A recent study by Rothschild et al. demonstrated that host genetics play a minor role in determining the microbiota composition of the gut [5]. Very similar findings were also previously reported for oral microbiota [6]. Rothschild et al. showed over 20% of the interpersonal microbiota variability is associated with factors related to diet, drugs, and anthropometric measurements. These studies provide evidence that it may be possible to alter the composition of an individual’s microbiota through changes in their environment and diet.

To date, many different mechanisms have been reported through which the microbiome influences our physiology and function. Microbiota are essential for digestion, the absorption of nutrients, and the regulation of endocrine, neurological, and immune systems [59]. They degrade polysaccharides, synthesize vitamins, and produce short-fatty chain acids such as butyric acid and propionic acid by fermentation [60]. Short fatty chain acids provide a vital source of energy for cells such as colonic epithelial cells and hepatocytes. Microbiota also play an important role in mucosal defense mechanisms. They create a more hostile environment for pathogenic bacteria by competing for nutrients, and are also essential to the development and optimal function of mucosal immune system defense mechanisms. Studies with germ-free animals have demonstrated significant defects in the gut immune response as well as reduced epithelial cell turnover [61]. Gut motility is also influenced and regulated by microbiota [62,63]. The concept of the gut–brain axis, which consists of a bidirectional interaction between gut microbiota and the peripheral and central nervous system affecting the behaviors and sensations of the host, is now well established [64,65]. An extensive review on the interplay between microbiota and human health and disease can be found in articles written by Clemente et al. [66] and Lin and Zhang [67].

The most represented phyla of bacterial microbiota in the human adult gut are Firmicutes (38.8%) and Bacteroidetes (27.8%), with Actinobacteria (8.2%) and Proteobacteria (2.1%) present in much smaller portions [68,69]. In a “stable” state, both microbiota and the gut provide bidirectional beneficial effects, creating a harmonious environment: a healthy gut. Dysbiosis is a term that is used to describe the disturbance and imbalance of a harmonious composition of gut microbiota, which can occur by events including a change in diet, enteric infections, the use of antibiotics, or abdominal surgery, and could cause undesirable symptoms. Dysbiosis, particularly a reduced biodiversity of microbiota, seems to be associated with several gastrointestinal diseases such as inflammatory bowel disease, but more recently its connection with systemic conditions such as Type I and II diabetes, autoimmune disorders, neurodegenerative diseases, obesity, and psychiatric episodes have also been reported [70,71,72,73,74]. The biggest question still to be answered and proven in this field of research is whether dysbiosis is a cause or an effect of these diseases.

More recently, there have also been studies showing an association between an individual’s microbiome and response to cancer therapy [75,76]. In these studies, an association was shown between patients with melanoma who had a greater diversity within their microbiome at the pre-treatment stage and a longer progression free-survival following immunotherapy (anti-PD1-therapy). Gopalakrishnan et al. also went on to show a significant positive correlation between tumor-infiltrating anti-tumor CD8^+^ lymphocytes and the abundance of *Faecalibacterium* genus, the *Ruminococcaceae* family and the Clostridiales order [75]. Also, a fecal microbiota transplant from treatment responders to germ-free mice resulted in greater tumor-infiltrating CD8^+^ lymphocytes, whereas fecal microbiota transplant from non-responders resulted in increased circulatory T-regulatory (Treg) lymphocytes and a blunted immune response. These findings suggest that dysbiosis could lead to alteration in the host’s immune system and systemically influence responsiveness to cancer treatment.

### 4.2. Gut Microbiome of Patients with Cancer

A study by Nam et al. investigated the microbiota of nine patients with gynecological cancer who underwent radiotherapy [77]. Their pre-radiotherapy microbiota were compared to that of six healthy controls, and although the top nine most abundant bacterial phyla were the same between cancer patients and healthy controls, the proportion of each phylum differed significantly between the two groups. Actinobacteria were 30 times more abundant in cancer patients compared to control individuals, whereas an abundance of Bacteroidetes, Fusobacteria, and Proteobacteria were lower in cancer patients. A distinct microbiota profile was also found in tumor tissue and in the nearby mucosa from patients with colorectal cancer in comparison to healthy controls [78]. The phylus Fusobacteria and the genus *Fusobacterium* were much more abundant in the colorectal cancer tissue with abundance of >7% compared to <0.5% in control samples. Euryarchaeota and Tenericutes were also significantly increased in the cancer tissue. Alterations in the several genera of phylum Firmicutes were seen in the cancer tissue, although the overall abundance of Firmicutes at the phylum level did not change. These studies are conducted in very small cohorts, and larger studies are required to confirm the findings. Whether a cancer-associated microbiota profile is a cause or an effect of the cancer is yet to be determined. However, it is important to acknowledge that the microbiome of individuals with cancer seems to differ from that of healthy individuals before they even receive bowel irradiation.

### 4.3. Effects of Radiation on Gut Microbiome and Incidence of Post-Radiation Diarrhea

Both animal and human studies suggest that irradiation results in significant changes in the gut microbiota of organisms [77,79,80,81,82,83]. The four clinical studies that examined the microbiota of patients with either gynecological or lower gastrointestinal tract cancers before and after radiotherapy all concluded that significant alterations in the microbiota profile were observed by irradiation [77,81,82,83]. However, there were discrepancies regarding the nature of these alterations between studies. Nam et al. showed that overall irradiation ameliorates interpersonal differences in microbiota profiles, making them more uniform amongst individuals [77]. A study by Manichanh et al. with 10 patients with gynecological cancers evaluated their data further and focused on different patterns of microbiota between patients who developed post-irradiation diarrhea and those who didn’t [81]. The authors found a difference in the microbiota profiles of the two cohorts in feces collected at the pre-irradiation stage. Furthermore, patients who developed diarrhea following irradiation showed a greater alteration in their microbiota profile compared to the patients who didn’t. In fact, the microbiota profiles in patients who did not develop diarrhea and healthy controls both maintained 60% of their bacterial composition during the period of radiotherapy. On the other hand, patients who developed diarrhea only maintained 29% of the similarity of their bacteria composition at the end of radiotherapy. Similarly, a study by Wang et al. of 11 patients with either gynecological or gastrointestinal cancers showed a reduction in the diversity of microbiota composition by irradiation in both radiation-induced diarrhea and non-diarrhea groups with a greater reduction in the diversity of microbiota composition in the diarrhea group [83]. Although these studies provide vital evidence for a link between the alterations in microbiota caused by radiotherapy and post-radiotherapy diarrhea, unfortunately they do not shed light on the mechanistic relationship between microbiota and radiation-induced bowel injury. They do not provide any objective evidence of bowel injury or inflammation in those who developed post-irradiation diarrhea.

An intriguing study conducted by Gerassy-Vainberg et al. demonstrated a potential pathogenic role of microbiota in radiation-induced alteration [84]. In this study, mice were exposed to localized internal radiation to induce proctitis, which resulted in a significant shift in their microbiota. This change was confined locally and correlated with tissue damage. The maximal microbiota change was observed at six weeks post-irradiation. Human epithelial cells (HT29 cell line) demonstrated the upregulation of IL-1β when co-cultured with fecal bacterial suspension collected from mice with radiation proctitis; in contrast, fecal bacteria from naïve control mice were unable to elevate cytokine release. This observation was tested in vivo by the delivery of fecal bacterial suspension from mice with radiation-induced proctitis or naïve control mice into germ-free mice by oral gavage, which were then exposed to irradiation. Germ-free mice that received bacterial suspension from mice with radiation proctitis showed greater bowel damage. The same results were found when germ-free mice with bacterial suspension were challenged with dextran sodium sulphate (DSS), which is a well-established compound to induce chemical colitis. These findings provide evidence for the first time that dysbiosis caused by radiation increases the bowel’s susceptibility to injury and seems to have a pathogenic role in driving radiation-induced bowel injury. Another study by Cui et al. showed that a sex-matched fecal transplant from naïve mice to radiation-exposed mice improved intestinal injury and survival, indicating that radiation-induced dysbiosis can be manipulated by fecal transplant to deliver therapeutic effect [85].

### 4.4. Effects of Probiotics on Acute Radiation-Induced Bowel Injury

Probiotics are defined as live microorganisms that confer a health benefit on the host when administered in adequate amounts [86]. Current evidence suggests that probiotics have a role in the prevention of *Clostridium difficile* infection, severe necrotizing enterocolitis in pre-term infants, and antibiotic-related diarrhea [87,88,89]. A beneficial effect of probiotics on post-radiotherapy gastrointestinal symptoms was shown as early as in 1988 when a twice-daily dosing of fermented milk containing >2 × 10^9^ live *Lactobacillus* was shown to reduce post-radiotherapy diarrhea amongst patients with gynecological cancers who received pelvic radiotherapy [90]. Studies investigating the effect of probiotics in radiation-induced gastrointestinal symptoms are difficult to evaluate, as they vary in the type of cancer patients recruited, radiotherapy modalities used, the presence or absence of concomitant chemotherapy use, end-point assessment, and the types of bacteria that are used as probiotics. Some have shown no benefit, whereas others have shown the significant benefit of probiotics in preventing post-radiation diarrhea [90,91,92]. A meta-analysis conducted in 2017 compiling six such independent randomized control studies investigating the effect of probiotics on post-radiotherapy diarrheal symptoms did conclude a beneficial effect with a significant reduction in the incidence of diarrhea by oral dosing of probiotics [93]. The incidence of post-radiation diarrhea was 175 out of 490 patients in the probiotic group compared to 246 out of 427 patients in control group (relative risk: 0.55; 95% CI: 0.34–0.88; *p* = 0.01). Interestingly, there was no statistical improvement in use of anti-diarrheal medications (relative risk: 0.68; 95% CI: 0.40–1.14; *p* = 0.14) or Bristol scale on stool form (relative risk: 0.64; 95% CI: 0.35–1.17; *p* = 0.14). All of the studies included in the meta-analysis examined the effect of probiotics on the prevention of diarrhea, and did not evaluate the difference in the duration of diarrhea between the probiotics and control groups. The dose regimes in the six studies varied from seven days prior to the radiotherapy to the day of the first dose of radiotherapy. Although there were variations in the type of bacteria contained in the probiotics that were used in the six studies, all of them contained *Lactobacillus* (two studies *Lactobacillus*, two studies *Lactobacillus* and *Bifidobacterium*, one study *Lactobacillus* and *Streptococcus*, one study *Lactobacillus*, *Bifidobacterium*, and *Streptococcus*). Although this is encouraging evidence, these human studies do not provide any mechanistic details or objective evidence of the beneficial effect of probiotics on radiation-induced bowel injury. Another study by Urbancsec et al. investigated the efficacy of a *Lactobacillus*-based probiotic as a rescue therapy for mild to moderate radiotherapy-induced acute diarrhea [94]. This did not show any significant benefit of the probiotic in reducing stool frequency.

### 4.5. Possible Mechanism of Intestinal Radioprotection Provided by Microbiome

An insight into the potential mechanistic effects of probiotics have been provided through a number of animal studies. It has previously been shown that a reduction of endotoxin levels, less severe histological bowel injury, and also attenuated Pseudomonas bacteremia could be induced by the administration of a *Lactobacillus*-based probiotic following exposure to radiation [95,96,97,98]. Another study in mice demonstrated reduced crypt cell apoptosis and greater crypt survival in *Lactobacillus rhamnosus*-treated animals after irradiation, which was dependent on a Toll-like receptor 2 (TLR2) and myeloid differentiation primary response 88 (MyD88)-dependent signaling pathway and caused the repositioning of cyclooxygenase-2 (COX2) expressing the mesenchymal stem cells from the lamina propria of villi near the crypt [99]. To date, there have not been any studies examining the effect of probiotics on the radiation-induced alteration in tight junction or mucosal permeability.

The possible way in which probiotics and the gut microbiome may prevent radiation-induced injury could be through the activation of Toll-like receptors (TLRs). TLRs are a family of proteins that are expressed by various immune cells such as macrophages, neutrophils, and dendritic cells, as well as epithelial cells. TLRs recognize conserved molecular patterns on pathogens, and upon doing, so trigger signaling pathways leading to cell proliferation, regulation of the cell cycle, and cytokine production, thus playing an important role in innate immunity. A pre-irradiation injection of the compound CBLB502 (Entolimod), a TLR5 ligand, has previously been shown to reduce the rate of apoptosis of intestinal crypts cells as well as cells within lamina propria in mice and primates [100]. Another study demonstrated that a pre-treatment with a TLR9 ligand in mice attenuated small bowel radiation injury through an MyD88-dependent signaling pathway [101]. Natural TLR5 and TLR9 ligands, bacterial flagellin and CpG (cytidine-phosphate-guanosine) DNA are typically found in bacteria and viruses, respectively. On the other hand, the activation of TLR3 and possibly TLR4 appears to exacerbate radiation injury to the bowel. Injection with TLR3 ligand Poly I:C, which is naturally found in viruses, lead to aggravated gastrointestinal symptoms after whole body irradiation. TLR3-knockout mice also demonstrated radioresistant properties by showing less apoptotic intestinal epithelial cells and also a greater number of radiation surviving crypts. Another ex vivo study using intestinal organoids from CCAAT/enhancer binding protein delta (C/EBPδ), a transcription factor with regulatory roles in inflammation and cell proliferation, knockout mice showed pre-treatment with TLR4 antagonist C34 reduced radiation-induced cell damage and death [102].

The mechanism by which alteration in the gut microbiome affects radiation-induced bowel injury is anticipated to be extremely complex, with dynamic changes in microbial communities interacting with various components of enteric systems, rather than a consequence of a single compound. Also, the above-mentioned in vivo studies with TLR ligands were performed with a systemic injection of TLR agonists and whole-body irradiation or localized radiation to the head and neck region. The number and composition of microbiota are different in the mouth and upper esophagus compared to the small and large intestine. Also, whole-body irradiation has an impact on the systemic immune system. Therefore, the radioprotective role of these compounds in radiation-induced bowel injury might be secondary to their effect on the systemic immune system. It is not known whether a similar result would be produced by the oral administration of TLR ligands and localized radiation to the abdominopelvic region. Furthermore, despite extensive evidence supporting the vital role of the microbiome in a healthy gut and emerging evidence supporting the radioprotective effect of bacterial compounds, such as the TLR5 ligand, it is well established that germ-free mice are more resistant to radiation-induced bowel injury than conventional mice colonized with microbiome [103,104]. Hence, it is speculated that a particular feature of some species of microbiota, at the right abundance, may provide a protective function in response to radiation.

### 4.6. Effect of Radiation-Induced Dysbiosis and Probiotics on Chronic Radiation-Induced Bowel Injury

To date, there have not been any animal or clinical studies that explore the association between radiation-induced dysbiosis and chronic radiation-induced bowel injury, nor any studies to investigate the effect of probiotics in the prevention of chronic radiation-induced bowel injury.

## 5. Conclusions

Increasing survival rates of cancer patients that have received abdominopelvic radiotherapy treatment has resulted in a rapid increase in the cohort of patients suffering from chronic radiation-induced bowel injury. Although recent studies have started to unravel the pathogenic mechanisms, our bench-side knowledge is a long way from being able to offer effective prevention or treatment. Current evidence suggests that the pathophysiology of acute and particularly chronic radiation-induced bowel injury is caused by a dynamic interplay of change in gut microbiota, epithelial cell damage and repair, endothelial injury and remodeling, fibrogenesis, alterations of the enteric nervous system, as well as inflammatory response orchestrated by the innate and adaptive immune system.

There is overwhelming evidence to show that the gut microbiome is significantly altered by radiation. This is probably to be expected, given the heterogeneity of an individual’s microbiota with varying degrees of radiosensitivity. There seems to be a specific pre-radiotherapy microbiota profile that is associated with risk of developing post-radiotherapy diarrhea among cancer patients. However, the highly individualized nature of each person’s microbiota profile, a complex interplay between host and environmental factors, and the heterogeneity of studies in this field make it almost impossible to currently conclude the microbiome profile that is associated with post-radiotherapy diarrhea. If such a profile is established, it could potentially be used as a predictive pre-therapy biomarker for post-radiotherapy acute diarrhea, and might be useful in delivering personalized radiotherapy regimes. Current evidence also suggests an association between the degree of change in microbiota caused by radiation and the likelihood of developing post-radiotherapy diarrhea. The reasons why radiotherapy alters one person’s microbiome more severely than another is still unknown. However, this seems to be an important factor in post-radiation diarrhea. Recently, the pathogenic property of radiation-induced dysbiosis in inducing bowel injury was shown for the first time in a mouse model, and has begun to shed light on how changes in the microbiome can influence bowel injury. Furthermore, another study showed a therapeutic effect of fecal transplant in improving radiation-induced bowel injury in mice. A first-ever clinical trial to investigate the efficacy of microbiota transplantation in the treatment of radiation-induced acute bowel injury is currently being conducted [105]. In this trial, patients who have endoscopically proven radiation-induced bowel injury will receive a selective microbiota transplant three times and be followed up for four weeks, at which point the efficacy will be assessed in various modalities, including endoscopy.

There is modest evidence of a beneficial effect of *Lactobacillus*-based probiotics in the prevention of radiation-induced acute diarrhea. Whether the use of probiotics ameliorates actual tissue injury is unknown, as the end-points of all of the studies are based solely on symptoms. Currently, probiotic use employs a trial and error approach of testing the effect of a particular strain of bacteria or combinations of bacteria chosen from thousands of potential bacterial strains, and the process is largely based on anecdotal evidence. If a mechanism through which bacterial strain(s) prevent post-radiation gastrointestinal symptoms was elucidated, more focused standardized studies could be conducted.

One area of research that is yet to be explored is a relationship between the gut microbiome and the chronic form of radiation-induced bowel injury. To date, studies in both animals and humans have all examined the link between the microbiome and acute diarrheal symptoms caused by radiation. The pathogenesis of acute radiation-induced bowel injury is different from chronic injury, and it is thought that the majority of these acute symptoms eventually subside to a considerable extent. Chronic radiation-induced bowel injury, on the other hand, is a progressive destructive disease, and patients who suffer from chronic radiation-induced bowel injury live with troublesome symptoms and co-morbidities, potentially indefinitely, with limited available therapeutic options. Evidence connecting the mechanistic role of the microbiome and systemic diseases, response to treatment, as well as acute gastrointestinal symptoms of abdominopelvic radiotherapy are starting to emerge. The potential therapeutic effect of the microbiome is also being discussed widely. Future research should examine the potential role of the microbiome in the prevention and treatment of chronic radiation-induced bowel injury and its associated morbidity.
